# COVID-19 pandemic: Insights into structure, function, and hACE2 receptor recognition by SARS-CoV-2

**DOI:** 10.1371/journal.ppat.1008762

**Published:** 2020-08-21

**Authors:** Anshumali Mittal, Kavyashree Manjunath, Rajesh Kumar Ranjan, Sandeep Kaushik, Sujeet Kumar, Vikash Verma

**Affiliations:** 1 Sriram Sameeksha, Jalahalli, Bangalore, India; 2 Suraj Darshan, Bangalore, Karnataka, India; 3 Department of Environmental Science, Central University of South Bihar, Gaya, India; 4 EduBooster Learnings, Vasundhara, Ghaziabad, India; 5 Centre for Proteomics and Drug Discovery, Amity Institute of Biotechnology, Amity University, Maharashtra, India; 6 Biology Department, University of Massachusetts, Amherst, Massachusetts, United States of America; University of Alberta, CANADA

## Abstract

Severe Acute Respiratory Syndrome Coronavirus-2 (SARS-CoV-2) is a newly emerging, highly transmissible, and pathogenic coronavirus in humans that has caused global public health emergencies and economic crises. To date, millions of infections and thousands of deaths have been reported worldwide, and the numbers continue to rise. Currently, there is no specific drug or vaccine against this deadly virus; therefore, there is a pressing need to understand the mechanism(s) through which this virus enters the host cell. Viral entry into the host cell is a multistep process in which SARS-CoV-2 utilizes the receptor-binding domain (RBD) of the spike (S) glycoprotein to recognize angiotensin-converting enzyme 2 (ACE2) receptors on the human cells; this initiates host-cell entry by promoting viral–host cell membrane fusion through large-scale conformational changes in the S protein. Receptor recognition and fusion are critical and essential steps of viral infections and are key determinants of the viral host range and cross-species transmission. In this review, we summarize the current knowledge on the origin and evolution of SARS-CoV-2 and the roles of key viral factors. We discuss the structure of RNA-dependent RNA polymerase (RdRp) of SARS-CoV-2 and its significance in drug discovery and explain the receptor recognition mechanisms of coronaviruses. Further, we provide a comparative analysis of the SARS-CoV and SARS-CoV-2 S proteins and their receptor-binding specificity and discuss the differences in their antigenicity based on biophysical and structural characteristics.

## Introduction

Before 2003, only 2 human coronaviruses—Human Coronavirus (HCoV)-229E and HCoV-OC43, causing mild illness—were known [1,2,3]. However, the emergence of Severe Acute Respiratory Syndrome Coronavirus (SARS-CoV) and Middle East Respiratory Syndrome Coronavirus (MERS-CoV) changed the view worldwide because coronaviruses can cause life-threatening infections [4,5,6]. The ongoing pandemic of a novel strain of coronavirus, Severe Acute Respiratory Syndrome Coronavirus-2 (SARS-CoV-2), is posing an unforeseen public health and economic threats worldwide. As of June 27, 2020, SARS-CoV-2 has infected more than 9.65 million people, with 491,115 deaths reported from 215 countries and territories [7], of which there are 2,407,590 confirmed cases of COVID-19 and 124,161 deaths in the United States of America alone [8]. Recombination, mutator alleles, and mutational robustness are some of the evolutionary mechanisms [9] that make coronaviruses capable of expanding their host ranges, including humans. Therefore, understanding the virology of the coronaviruses at a structural level is of utmost importance because the health threats from these zoonotic viruses are constant and long-term.

Coronaviruses are large, enveloped, positive-stranded RNA viruses responsible for infecting a wide variety of mammalian and avian species [10]. These viruses contain spike-like projections of glycoproteins on their surface, which appear like a crown under the electron microscope; hence, they are referred to as coronaviruses. The coronavirus genome encodes several structural and nonstructural proteins. The structural proteins are responsible for host infection [11], membrane fusion [12], viral assembly [13], morphogenesis, and release of virus particles [14], among other functions, and the nonstructural proteins (nsps) facilitate viral replication and transcription [15,16]. The membrane (M), the envelope (E), and the spike protein (S) make up the structural proteins and are associated with the envelope. Among these structural proteins, the trimeric S proteins protrude from the virus envelope and are the key machinery that facilitates virus entry into the host cell [10,17].

The S proteins are clove-shaped, type-I transmembrane proteins and have 3 segments: a large ectodomain, a single-pass transmembrane, and an intracellular tail. The ectodomain of S proteins consist of the S1 subunit, containing a receptor-binding domain (RBD), and the membrane-fusion subunit (S2). The host-cell receptor recognition by the RBDs on S proteins is the initial step of viral infection, and the binding interactions between the coronavirus spike and its receptor is one of the most critical factors for host range and cross-species transmission. Human coronaviruses recognize a variety of host receptors; specifically, HCoV-229E recognizes human aminopeptidase N (hAPN) [18], MERS-CoV binds dipeptidyl peptidase-4 (DPP4) [19], HCoV-OC43 and HCoV-HKU1 bind certain types of O-acetylated sialic acid [20], and HCoV-NL63 and SARS-CoV recognize angiotensin-converting enzyme 2 (ACE2) [21,22]. Recent structures, along with functional studies, have suggested that the SARS-CoV-2 S proteins utilize ACE2 and Transmembrane Serine Protease 2 (TMPRSS2) for host-cell entry, which are very similar to the mechanisms exploited by SARS-CoV [23]. See the “Structure, function, antigenicity, and hACE2 receptor recognition by the SARS-CoV-2 S glycoprotein” section of this review for detailed information on the mechanism of coronavirus cell entry mediated by the viral S glycoproteins. The S proteins, common among all coronaviruses, are a major target for eliciting antibodies; therefore, structural and molecular details of S protein and its interactions with cognate receptors would be vital in developing vaccines and antiviral drugs against SARS-CoV-2.

In this review, we discuss the coronavirus classification, details of SARS-CoV-2 emergence, morphology, and key virulence factors. We specifically explain the structure of RNA-dependent RNA polymerase of SARS-CoV-2 and its significance in drug discovery. Further, the structure, function, and antigenicity of S glycoproteins and their interactions with human ACE2 (hACE2) receptor are discussed.

## Emergence of SARS-CoV and SARS-CoV-2

**Fig 1:**
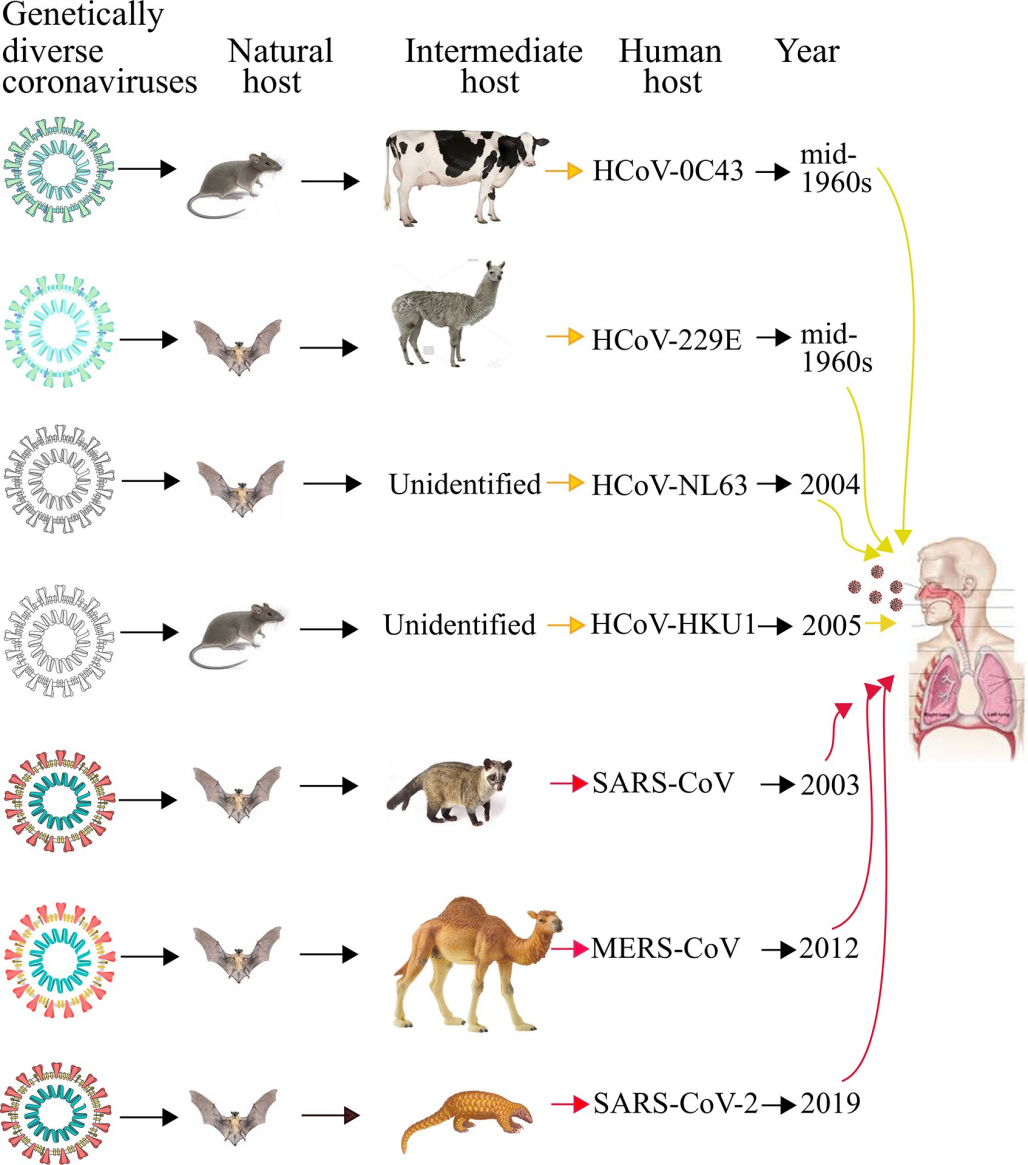
Origin and transmission of pathogenic HCoVs. Yellow and red arrows indicate mild and severe infections in humans, respectively. The figure is inspired from Jie Cui and colleagues [46], and the illustrations of coronaviruses (left) are adapted from “Desiree Ho, Innovative Genomics Institute,” available at https://innovativegenomics.org/free-covid-19-illustrations/. HCoV, Human Coronavirus; MERS-CoV, Middle East Respiratory Syndrome Coronavirus; SARS-CoV, Severe Acute Respiratory Syndrome Coronavirus; SARS-CoV-2, SARS-CoV, Severe Acute Respiratory Syndrome Coronavirus-2.

In November 2002, SARS began spreading from the Guangdong province of Southern China, but its reservoir was unknown. In the past, Nipah and Hendra, both zoonotic viruses, originated from bats, and this motivated researchers to find whether bats are the natural reservoirs of SARS-CoV [24,25]. In 2005, 2 research groups independently reported that bats (horseshoe bats in particular) are the natural host of genetically diverse coronaviruses and closely related to those responsible for the SARS outbreak [26,27]. These viruses were termed SARS-like coronaviruses, and they displayed considerable genetic similarities to SARS-CoV isolated from humans or civets. This suggested that the virus responsible for SARS outbreak was a member of the SARS-like coronaviruses group [26]. In Saudi Arabia, MERS-CoV emerged in 2012, when humans were infected through direct or indirect contacts with infected dromedary camels. However, genome analysis suggested that MERS-CoV might have also originated in bats and was transmitted to camels in the distant past [28] (Fig 1).

**Fig 2:**
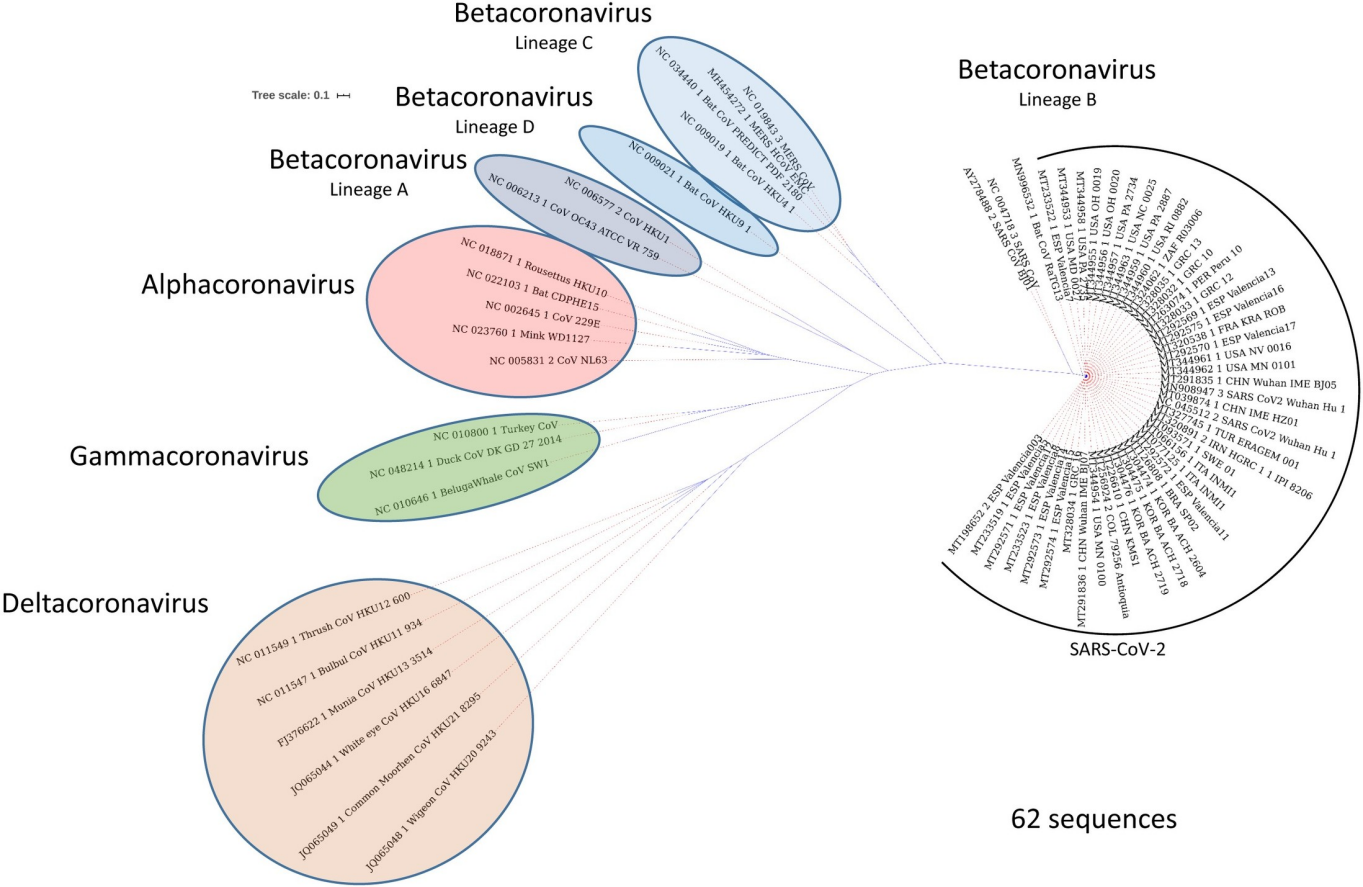
Phylogenetic relationships in the Coronavirinae subfamily. The subfamily is formed by 4 genera: *Alphacoronavirus*, *Betacoronavirus* (lineages A, B, C, and D), *Gammacoronavirus*, and *Deltacoronavirus*. We randomly picked 62 SARS-CoV-2 genome sequences, representing 15 different countries, together with other Coronavirinae subfamily members. The phylogenetic tree was created using NgPhylogeny.fr tool. The analysis indicates that SARS-CoV-2 has a close relationship with bat coronavirus RaTG13 and SARS-CoV; therefore, it is classified as a new member of the lineage B *Betacoronavirus*. SARS-CoV, Severe Acute Respiratory Syndrome Coronavirus; SARS-CoV-2, Severe Acute Respiratory Syndrome Coronavirus-2.

In December 2019, patients with severe pneumonia cases of unknown cause were reported in Wuhan, China, and a novel coronavirus strain was detected from the lower respiratory tract of 4 patients [29]. Viruses were isolated from these clinical samples, and their genomes were analyzed by deep sequencing [30,31,32]. Phylogenetic analysis of 2019-novel coronavirus (2019-nCoV) genomes and other coronaviruses were used to establish the evolutionary history and infection sources. Interestingly, this indicated that 2019-nCoV (GenBank: MN908947.3) shares about 96% nucleotide sequence identity to bat coronavirus RaTG13 (GenBank: MN996532.1), with 79.5% and 55% identity to SARS-CoV BJ01 (GenBank: AY278488.2) and MERS-CoV HCoV-EMC (GenBank: MH454272.1), respectively, and belongs to the same family of viruses that caused SARS and MERS (Fig 2 and S1 Fig). This suggests that bats are possibly the hosts of 2019-nCoV origin, and it might have been transmitted either directly from bats or through an unknown intermediate host to infect humans [29,33,34,35]. Despite high sequence similarities, a few notable and conserved variations arose in 2019-nCoV genomes that were not previously seen in betacoronaviruses. These notable features, which establish this virus as different from SARS-CoV and SARS-like coronaviruses, are (i) multiple mutations in the RBDs of S protein that may interact with ACE2 receptor, (ii) a polybasic furin-like protease site (RRAR/S) at the boundary of S1/S2 subunits rather than the single arginine observed in SARS-CoV, and (iii) the addition of 3 predicted O-linked glycans flanking the protease site [36,37]. Of note, a furin-like protease site is a signature of several highly pathogenic avian influenza viruses and pathogenic Newcastle disease virus [38,39].

Originally, this virus was called 2019-nCoV, but later the International Committee on Taxonomy of Viruses on February 11, 2020 officially named it SARS-CoV-2 because of its genetic similarity to SARS-CoV [33]. SARS-CoV-2 causes respiratory illness, and WHO named this illness COVID-19. It is contagious, primarily transmitted among people through respiratory droplets and contact routes [40,41], and more than 13 million COVID-19 cases are confirmed worldwide (as of July 14, 2020). Initially, WHO declared the COVID-19 outbreak a public health emergency of international concern and later confirmed it as a pandemic on March 11, 2020 [42].

## Classification of coronaviruses

**Fig 3:**
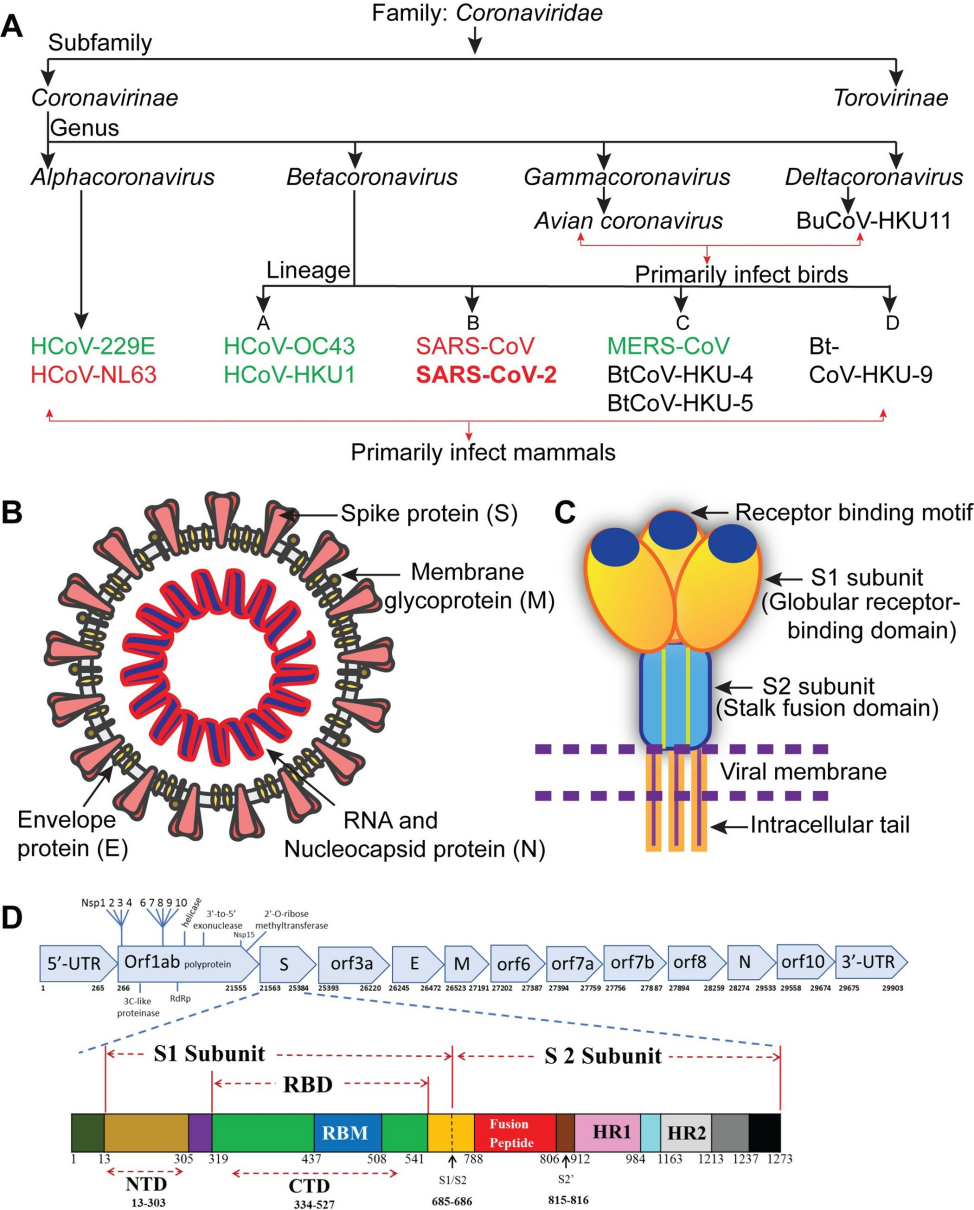
Classification and structure of coronavirus. (A) Classification of coronaviruses: the 7 known HCoVs are shown in green and red. HCoVs in red bind the host receptor ACE2. (B) Schematic of the SARS-CoV-2 structure; the illustration of the virus is adapted from “Desiree Ho, Innovative Genomics Institute,” available at https://innovativegenomics.org/free-covid-19-illustrations/. (C) Cartoon depicts key features and the trimeric structure of the SARS-CoV-2 S protein. (D) Schematic of SARS-CoV-2 genome (top) and S protein (bottom); annotations are adapted from NCBI (NC_045512.2) and Expasy (https://covid-19.uniprot.org/uniprotkb/P0DTC2), respectively. ACE2, angiotensin-converting enzyme 2; CTD, C-terminal domain; E, envelope; HCoV, Human Coronavirus; HR1/2, heptad repeat 1/2; M, membrane; N, nucleocapsid; Nsp, nonstructural protein; NTD, N-terminal domain; orf, open reading frame; RBD, receptor-binding domain; RBM, receptor-binding motif; RdRp, RNA-dependent RNA polymerase; S protein, spike protein; SARS-CoV-2, Severe Acute Respiratory Syndrome Coronavirus-2; UTR, untranslated region.

The coronavirus study group of the International Committee on Taxonomy of Viruses has classified coronaviruses under the family Coronaviridae, subfamily Coronavirinae. Based on genotypic and serological characterization, Coronavirinae is divided into 4 genera: *Alphacoronavirus*, *Betacoronavirus*, *Gammacoronavirus*, and *Deltacoronavirus* [43,44,45,46] (Fig 3A). Only 6 HCoV species that cause human disease were known before December 2019. Four of them cause common cold symptoms in immunocompromised individuals: these are HCoV-229E and HCoV-OC43, first identified in the mid-1960s [1,2,3]; HCoV-NL63, first identified in 2004 [47,48]; and HCoV-HKU1, first identified in 2005 [49]. The other 2 strains, which cause fatal illness, are SARS-CoV, first identified in 2003 [4,5], and MERS-CoV, first identified in 2012 [6]. SARS-CoV-2 has 96% nucleotide sequence identity to bat coronavirus RaTG13, a SARS-like coronavirus; therefore, it belongs to *Betacoronavirus* genera (Fig 2 and S1 Fig).

Forsters and colleagues [50] performed a phylogenetic network analysis of 160 complete SARS-CoV-2 genomes sampled from across the world to understand the evolution of this virus in humans and infection sources. They sorted these closely related genomes into 3 lineages, namely A, B, and C, based on amino acid changes. Lineage A was named for the original bat coronavirus that caused COVID-19, but surprisingly, it was not the dominant virus type in Wuhan. The A and C types were found largely in the Americas and Europe, respectively, whereas the B type was mostly prevalent in East Asia and had acquired mutations before spreading outside East Asia. The lineage C differs from its parent lineage B by a mutation at amino acid position 26,144 and was prevalent in France, Italy, Sweden, England, California, Brazil, Singapore, Hong Kong, Taiwan, and South Korea but absent from mainland Chinese samples. This kind of phylogenetic classification has potential for accurately tracing the infection routes and will prove helpful in designing treatments and vaccine development [50].

## Morphology, genomic structure, and key viral factors of SARS-CoV-2

Coronaviruses are nonsegmented, enveloped viruses with single-stranded RNA (ssRNA) ranging between 26 to 32 kb in length. At this length, the coronavirus genome is the largest among RNA viruses. Electron microscopy (EM) of negative-stained SARS-CoV-2 particles revealed their spherical shape, with the diameter ranging from 60–140 nm and an outer surface studded with distinctive 9- to 12-nm–long spikes that gave virions the appearance of a solar corona [29] (Fig 3B). The observed morphology of SARS-CoV-2 is consistent with other members of the Coronaviridae family.

SARS-CoV-2 Wuhan-Hu-1 isolate (GenBank: MN908947.3) was among the first complete genome of the virus strains to be sequenced and comprises a 29,903-bp–long RNA. It is 5′-capped and 3′-polyadenylated, consists of 2 flanking untranslated regions (UTRs), and contains several open reading frames (orfs) that encode multiple proteins. The genome is arranged in the order of a noncoding 5′-UTR–replicase genes (orf1ab)–structural proteins (S, E, M, and N) and accessory proteins–noncoding 3′-UTR [51] (Fig 3D). Notably, it lacks the hemagglutinin-esterase gene, which is a common feature of lineage A *Betacoronaviruses* [31]. The orf1a/b, located at the 5′-end of the genome, is the largest orf, and it encodes 15 nsps (nsp1–10 and nsp12–nsp16) [52]. Briefly, the orf1a/b has overlapping orfs and produces 2 polypeptides, pp1a and pp1ab, because of ribosomal frameshifting. The virus genome encodes 2 cysteine proteases, a papain-like protease (PL2pro) or nsp3 and a 3C-like protease (3CLpro) or nsp5. These proteases cleave pp1a and pp1ab polypeptides into 15 nsps. Specifically, PL2pro is responsible for cleaving between nsp1|2, nsp2|3, and nsp3|4 sites, and the 3CLpro cleaves at the LQ↓SAG sites to produce nsp4 through nsp16 [31,53]. RNA-dependent RNA polymerase (nsp12) in complex with nsp7, nsp8, helicase (nsps13), and exonuclease (nsp14) are critical enzymes among these nsps responsible for the transcription and viral RNA replication.

The 3′-terminus of the SARS-CoV-2 genome contains 4 structural proteins that are responsible for virus–host cell receptor binding, virion assembly, morphogenesis, and release of virus particles from the host cell. The E protein of SARS-CoV-2 is the smallest of all structural proteins found in the viral membrane and localizes to the endoplasmic reticulum and Golgi complex in the host cells [54]. The E protein, along with M and N, is known to facilitate virus-like particle formation [14]. The M glycoprotein is a transmembrane protein located in the viral membrane and is the most abundant structural protein in a virion, almost a hundred times more abundant than E protein. The M protein plays a major role in the viral assembly along with E and N proteins [13,14,55]. The N protein is responsible for packaging the viral genome RNA (gRNA) into a helical ribonucleocapsid (RNP). SARS-CoV-2 also has 8 accessory proteins derived from subgenomic RNA: 3a, 3b, 6, 7a, 7b, 8b, 9b, and orf14 (based on the National Center for Biotechnology Information [NCBI] annotation NC_045512.2, and [52]), and they are distributed among the structural genes [51,52,56].

Phylogenetic-tree–based analysis of the whole genomes and individual genes suggest that SARS-CoV-2 is closer to SARS-like bat coronaviruses than to SARS-CoVs. Specifically, the S gene of SARS-CoV-2 is closer to SARS-like bat coronaviruses, although the 3a and 8b accessory genes are closer to SARS-CoVs [52,57]. In a recent study based on available genomic sequences, it was observed that the SARS-CoV-2 (106-sequence) genome has a much lower mutation rate and genetic diversity than SARS-CoV (39 sequences), and in particular, the S-protein–coding gene is relatively more conserved than other protein-encoding genes [58].

### Structure of the SARS-CoV-2 RNA-dependent RNA polymerase complex

**Fig 4:**
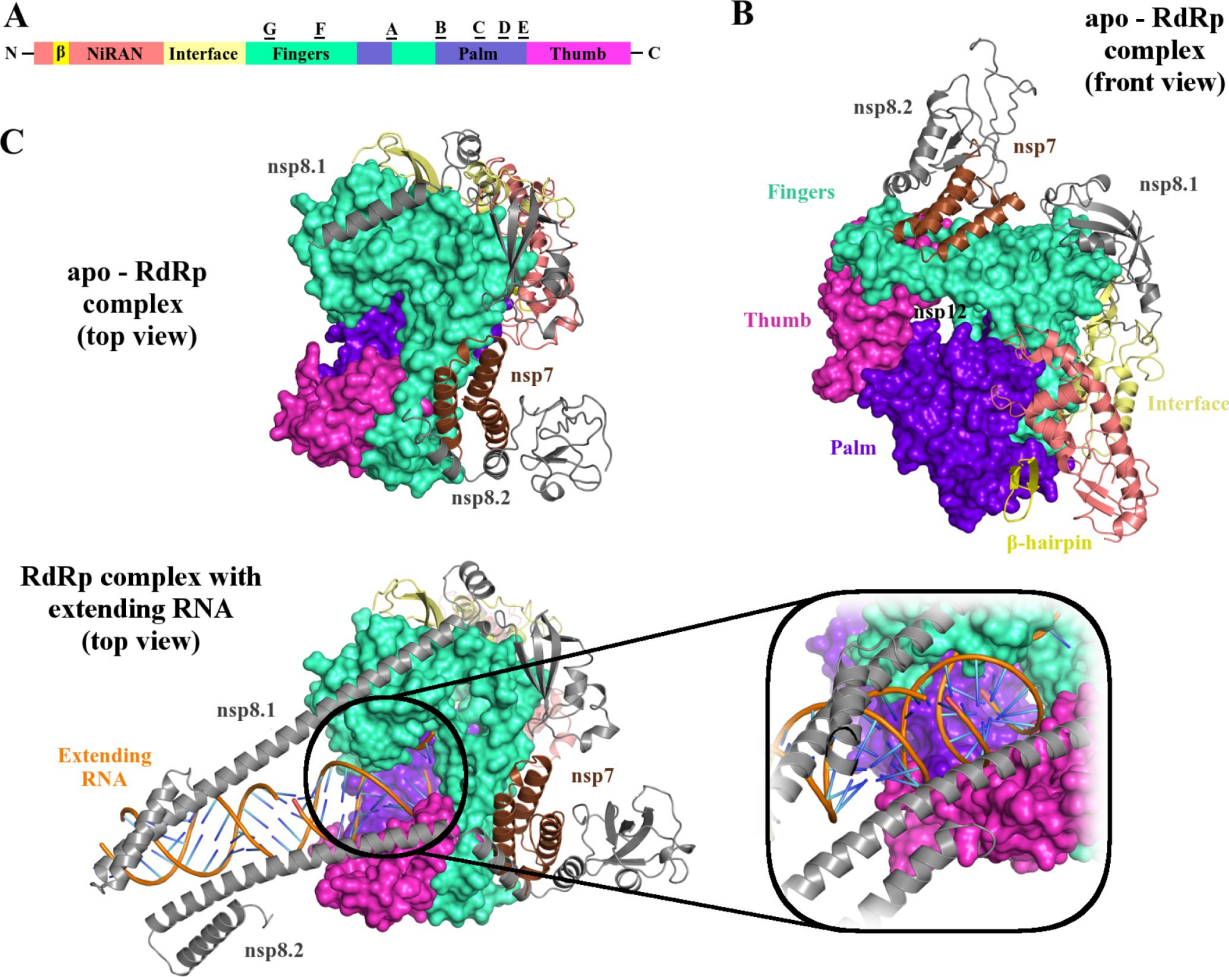
Cryo-EM structure of RdRp of SARS-CoV-2. (A) The domain architecture of RdRp or nsp12 of SARS-CoV-2 is subdivided into NiRAN, interface, fingers, palm, and thumb subdomains; A–G indicate conserved motifs. (B) The cryo-EM structure of apo-RdRp complex (shown as front view, PDB: 7BV1) consists of nsp12, nsp7 (brown), and 2 chains of nsp8 (nsp8.1 and nsp8.2, both in gray). The nsp8.1 interacts directly with nsp12, whereas the nsp8.2 binds to nsp7, which in turn interacts with nsp12. The RNA template is expected to enter the active site, which is formed by motifs A and C through a groove clamped by motifs F and G. Motif E and the thumb subdomain support the primer strand. The RdRp subdomain color scheme is according to Fig 4A. (C) The cryo-EM structure (in top view) of the RdRp complex bound to RNA (PDB: 6YYT) shows 2 chains of nsp8 stabilizing the extending RNA with their alpha helices. The apo-RdRp complex structure (PDB: 7BV1) is shown for comparison. The active site is expanded to show the RNA molecules coming out of the groove formed by the finger and the thumb subdomains. The figures were prepared using Pymol. cryo-EM, cryo-electron microscopy; NiRAN, nidovirus RdRp-associated nucleotidyltransferase; PDB, Protein Data Bank; RdRp, RNA-dependent RNA polymerase; SARS-CoV-2, Severe Acute Respiratory Syndrome Coronavirus-2.

Coronaviruses use an RNA-dependent RNA polymerase (RdRp) complex for replication of their genome and transcription of their genes [16]. The SARS-CoV-2 RdRp complex is composed of a catalytic subunit nsp12 and two accessory subunits nsp7 and nsp8, which increase RdRp template binding and processivity [59]. The mechanism of replication and inhibition of SARS-CoV-2 RdRp has been elucidated by several groups using cryo-EM structures of the RdRp–nps7–nsp8 complex [15], its complex with RNA [60], and Remdesivir [61]. The overall structure of the SARS-CoV-2 nsp12–nsp7–nsp8 complex highly resembles that of SARS-CoV, with a global root mean-square deviation (RMSD) of approximately 1 Å for the α-carbon atoms [15,62]. The SARS-CoV-2 RdRp complex structure reveals that the nsp12 core catalytic subunit is bound to a heterodimer of nsp7–nsp8 and an additional nsp8 subunit at a different binding site (Fig 4A–4C) [62]. The N-terminus of nsp12 contains nidovirus RdRp-associated nucleotidyltransferase (NiRAN) domain followed by an interface domain and a C-terminal RdRp domain (Fig 4B) [15]. The RdRp domain includes 7 conserved motifs (A–G), which are distributed in the finger, palm, and thumb subdomains (Fig 4A and 4C). The palm subdomain is formed by 5 conserved motifs A–E; motif C contains a critical SDD sequence (“Ser-Asp-Asp” residues 759–761), which forms the catalytic active center. Both D760 and D761 coordinate with 2 magnesium ions at the catalytic center. The F and G motifs are located within the finger subdomain and direct the template strand RNA into the active site, and the thumb subdomain intersects the extensions from the finger subdomain to hold the first turn of RNA [59,60,61,62]. The residues involved in RNA binding as well as forming the catalytic active site are highly conserved among different RNA viruses, which highlights the conserved mechanism of genome replication used by RdRp [61].

The RNA polymerase of the viruses is an established target for inhibiting the viral replication and has pre-established values for clinical engagements by the broad-spectrum nucleotides, such as prodrug remdesivir. These drugs have shown therapeutic efficacies against several viruses from different families, including Ebola, Nipah, MERS, and SARS-CoV [63,64]. The cell-based studies in Vero E6 cells (American Type Culture Collection [ATCC]-1586) have shown that Remdesivir is able to potently block SARS-CoV-2 viral infections at very low concentrations (EC_50_  =  0.77 μM) in vitro [65]. The cryo-EM structure of the RdRp–Remdesivir complex suggests that Remdesivir inhibits the viral RdRp activity through nonobligate RNA chain termination, a mechanism that converts the prodrug to the active drug in the triphosphate form [61]. Besides Remdesivir, Flavipiravir, Ribavirin, Galidesivir, and EIDD-2801 have been shown to inhibit SARS-CoV-2 replication in cell-based assays. Specifically, EIDD-2801 is 3–10 times more potent than Remdesivir in blocking SARS-CoV-2 replication [66]. The cryo-EM structure of the RdRp–Remdesivir complex (Protein Data Bank [PDB]: 7BV2) provides the mechanism of Remdesivir binding, as well as a blueprint for designing more potent antiviral therapeutics to combat the vicious infection of SARS-CoV-2 [61].

## Structure, function, antigenicity, and hACE2 receptor recognition by the SARS-CoV-2 S glycoprotein

**Fig 5:**
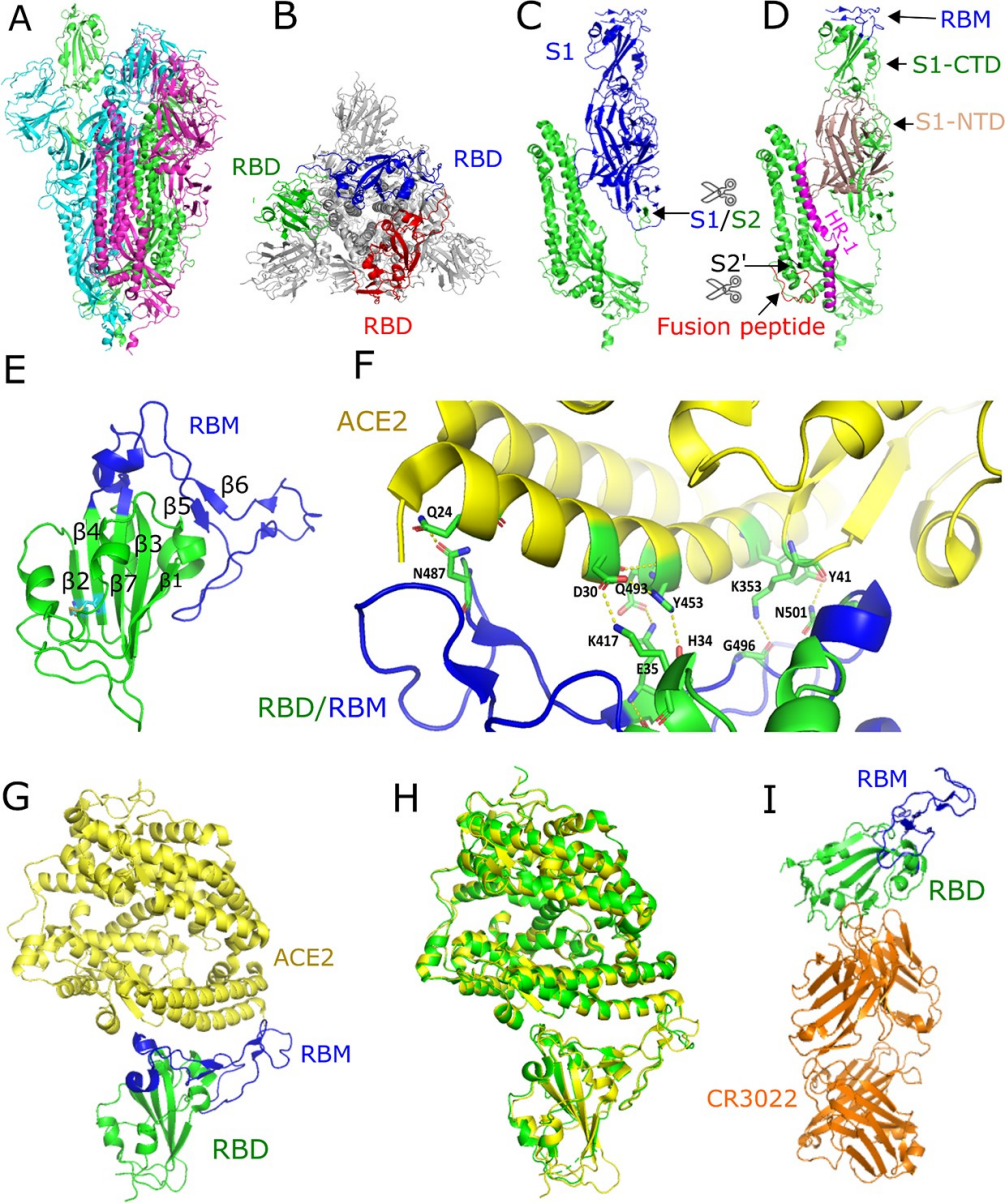
Structure of the SARS-CoV-2 S protein alone and in complex with ACE2 receptor. (A) Side view of the trimeric SARS-CoV-2 S ectodomain in the prefusion state (PDB: 6VSB). The protomer in green is in the “up” conformation, and the other 2 protomers in red and cyan are in “down” conformation. (B) Top view of the trimeric S protein showing RBDs in red, blue, and green on each protomer. (C) Structure of a single protomer showing the receptor-binding subunit S1 (blue) and the membrane-fusion subunit S2 (green). The furin-like protease site at the boundary of S1/S2 subunits is depicted. (D) The S1 subunit showing the RBM in the CTD region (blue) and the NTD region (brown). The S2 subunit showing the fusion peptide (red), second cleavage site S2′ (black), and HR1 (pink). (E) Structure of the RBD, core subdomain (green), and RBM (blue) (PDB: 6LZG). (F) SARS-CoV-2-RBD:ACE2 receptor polar interface shown by specific residues. (G) Structure of the SARS-CoV-2-RBD in complex with ACE2 receptor (PDB: 6LZG). (H) Structural similarity between the SARS-CoV-RBD:hACE2 (green) and SARS-CoV-2-S-CTD:hACE2 (yellow) complexes. (I) Crystal structure of the SARS-CoV-2-RBD (green) in complex with a monoclonal antibody CR3022 (orange). The RBM and CR3022 binding sites do not overlap and are distantly located on the RBD (PDB: 6W41). The figures were prepared using Pymol. ACE2, angiotensin-converting enzyme 2; CTD, C-terminal domain; hACE2, human ACE2; HR1, heptad repeat 1; NTD, N-terminal domain; PDB, Protein Data Bank; RBD, receptor-binding domain; RBM, receptor-binding motif; S protein, spike protein; SARS-CoV, Severe Acute Respiratory Syndrome Coronavirus; SARS-CoV-2, Severe Acute Respiratory Syndrome Coronavirus-2.

The S protein is a multifunctional molecular machine that plays key roles in the early steps of viral infection by interacting with host susceptibility factors, including receptors and proteases. These interactions subsequently infect human cells, which contain hACE2 transmembrane proteins [67]. The SARS-CoV-2-S is a transmembrane glycoprotein composed of S1 regions containing the NTD and CTD, S2, a transmembrane region, and a short cytoplasmic domain (Fig 3C and 3D). Both cryo-EM and crystallographic methods have been used to determine multiple structures of the SARS-CoV-2 S protein alone, including the ectodomain of S protein (SARS-CoV-2-S), RBD (SARS-CoV-2-RBD), or in complex with full-length hACE2 or soluble hACE2/B°AT1, in a very short time. These structural studies have enabled us to understand the molecular basis of SARS-CoV-2 entry into human cells displaying ACE2 receptors [17,68,69,70]. Several structures of SARS-CoV-2-S were observed in multiple states (the prefusion, closed, and partially open conformations and in complex with hACE2 receptor) with the RBDs either in an “up” or “down” conformation (Fig 5A and 5B). Of note, to engage the hACE2 receptor, the RBDs of S1 undergo hinge-like movements that either expose or hide the receptor-binding regions and these conformations are referred to as “up” (receptor-accessible) or “down” (receptor-inaccessible) conformations, respectively. SARS-CoV-2-S structures show that the protein adopts a clover-shaped homotrimeric structure, with 3 S1 heads that recognize a cognate cell-surface receptor and a membrane-anchored trimeric S2 stalk that contains the fusion machinery and is primarily α-helical [17] (Fig 5C and 5D). In the prefusion conformation of SARS-CoV-2-S protein, the RBDs rest above the trimeric S2 stalk, exhibiting 2 protomers in the “down” conformation and 1 protomer in the “up” conformation, which is a receptor-accessible state required for binding to an hACE2 receptor [17]. Overall, the SARS-CoV-2-S ectodomain resembles the closely related SARS-CoV-S structure with an RMSD of 3.8 Å over 959 Cα atoms, with a high degree of structural homology when individual domains of SARS-CoV-S and SARS-CoV-2-S were aligned [17].

### SARS-CoV-2-RBD interactions with hACE2 receptor

Multiple structures of SARS-CoV-2-RBD in complex with either full-length hACE2 or soluble hACE2 have shown that the extracellular peptidase domain (PD) of ACE2 recognizes the RBDs of S protein mainly through polar interactions [68,69]. Similar to other betacoronaviruses, the SARS-CoV-2-RBD structure suggested that it contains 2 subdomains: a core subdomain containing a twisted 5-stranded antiparallel β-sheet (β1, β2, β3, β4, and β7) with a conserved disulfide bond between β2–β4. The other subdomain is the receptor-binding motif (RBM), located between β4 and β7 strands as an extended insertion (Fig 5E).

The RBM forms a gently concave surface that accommodates the N-terminal α-helix of the hACE2 and a series of hydrophilic residues that form a solid network of H-bond and salt bridge interactions (Fig 5F). In brief, strong polar contacts include CTD residues A475, N487, E484, and Y453 that interact with S19, Q24, K31, and H34 of the α1 helix of hACE2, respectively [11]. In addition, residues Q498, T500, and N501 on the bulged loop form a network of H-bonds with Y41, Q42, K353, and R357 from hACE2 [68]. Thus, overall virus–receptor interactions are dominated by polar contacts mediated by hydrophilic residues [11,68,69] (Fig 5G).

### Comparison of the SARS-CoV-2-RBD and SARS-CoV-RBD interactions with hACE2 receptor

The majority of the secondary structure elements shared between SARS-CoV-RBD (PDB: 2AJF) and SARS-CoV-2-RBD (PDB: 6LZG, 6M17) are well superimposed, with an RMSD of 0.475 Å over 128 Cα atoms, with the exception of the receptor-binding loop. Interestingly, these structures revealed that the majority of binding sites of SARS-CoV-RBD in hACE2 also overlap with the SARS-CoV-2-RBD–binding sites, suggesting that the SARS-CoV-2-RBD:hACE2 complex is strikingly similar to the SARS-CoV-RBD:hACE2 structure with an RMSD of 0.431 Å over 669 Cα atoms (Fig 5G and 5H). However, despite the overall similarity, a number of sequence variations were observed at the binding interface that may account for the difference in the affinities for hACE2 receptors. The detailed comparison of the receptor-binding interfaces suggested that the SARS-CoV-2-RBD:hACE2 complex (PDB: 6VW1, 6M17) has larger buried surface areas (1,773 Å^2^ versus 1,686 Å^2^), has additional contacts (21 versus 17) and more van der Waals interactions (288 versus 213), as well as more H-bonds (16 versus 1), than the SARS-CoV-RBD:hACE2 (PDB: 2AJF) complex [69]. Additionally, residue F486 in SARS-CoV-2-RBD forms stronger aromatic–aromatic interactions with Y83 of hACE2 than I472 of SARS-Co-V-RBD. Residue E484 in the SARS-CoV-2-RBD also forms stronger ionic interactions with K31 compared to P470 of SARS-CoV-RBD [69]. A SARS-CoV-2 sample collected from the state of Kerala in India on January 27, 2020, revealed an Arg408 → Ile408 mutation in the SARS-CoV-2-S protein (GenBank: MT012098.1), which otherwise is a strictly conserved residue in SARS-CoV, SARS-CoV-2, and bat SARS-like CoVs. Residue R408 is located near to the binding interface of both the SARS-CoV-2-RBD:hACE2 (PDB: 6VW1) and SARS-CoV-RBD:hACE2 (PDB: 2AJF) complexes but appears not to be interacting directly with hACE2 in either case. However, R408 does form an H-bond (3.3 Å) with the glycan attached to N90 from hACE2, thus potentially contributing to the higher affinities observed for SARS-CoV2-RBD:hACE2 interactions than the SARS-CoV-RBD:hACE2 complex, in which the corresponding R395 is located relatively further away (6.1 Å) from N90 of hACE2. The Arg408 → Ile408 mutation that emerged in SARS-CoV-2 strain (GenBank: MT012098.1) suggested that the loss of H-binding capacity could potentially reduce hACE2 binding affinity. The equilibrium dissociation constants (K_D_) for hACE2 interacting with the S proteins have indicated that the binding affinity of SARS-CoV-2-S is several-fold higher than that of SARS-CoV [11,17,69].

Using cryo-EM, the structure of full-length hACE2 in complex with SARS-CoV-2-RBD and B°AT1 (neutral amino acid transporter) was determined. This structure revealed that the hACE2:B°AT1 complex is assembled as a dimer of heterodimers, in which the collectrin-like domain of hACE2 drives homodimerization (PDB: 6M17) [68]. The SARS-CoV-2-RBD is recognized by the extracellular PD of ACE2 as described previously. Further, it demonstrates that a homodimeric ACE2 can accommodate 2 S protein trimers, each through a monomer of ACE2 [68]. Interestingly, a superimposition of the ternary complex on the RBD in the “down” conformation has indicated that the PD clashes with the S protein, whereas in the “up” conformation (PDB: 6VSB), no clashes are observed. This suggests that the “up” conformation of the RBD is a receptor-accessible state and is essential for the ACE2-receptor binding. Taken together, the overall interface between SARS-CoV2-RBD:hACE2 is very similar to the previously known SARS-CoV-RBD:hACE2 interface and is dominated by the polar interactions, as reported by different investigations [11,68,69]. These observations further suggest that SARS-CoV-2-RBD has increased atomic interactions with hACE2, which results in higher affinities compared with the SARS-CoV-RBD:hACE2 complex, which might be one of the reasons for enhanced human-to-human transmission of SARS-CoV-2.

### SARS-CoV-2 exhibits distinct epitope features on the RBD from SARS-CoV

Numerous binding and neutralization epitopes have been identified on the S protein of coronaviruses, making the S protein an essential target for vaccine design [71,72,73]. Soon after the emergence of COVID-19 pandemic, some of the initial efforts were focused on screening SARS-CoV-S–specific antibodies to find neutralizing antibody/antibodies for vaccine and drug development against SARS-CoV-2. The hypothesis behind these studies was based on significant sequence as well as structural similarities, and moreover, both viruses bind to the same receptor with overlapping epitopes. Therefore, it was expected that SARS-CoV–specific antibody/antibodies alone or in combination can interfere or even inhibit SARS-CoV-2 and hACE2 receptor interactions.

It has been shown in vitro, as well in animal models, that monoclonal antibodies such as 80R [74], CR3014 [75], S230.15 [76], and m396 [76] can block binding of the S1 domain and hACE2 receptors by potently neutralizing SARS-CoV. However, CR3022 [77] alone did not show neutralization, but the mixture of CR3022 and CR3014 both showed neutralization of SARS-CoV in a synergistic fashion by recognizing different epitopes on the RBDs [75]. Of note, a report suggests that CR3022 can also neutralize SARS-CoV alone [78]. Interestingly, researchers from China tested several published SARS-CoV–specific monoclonal antibodies and found that CR3022 can bind to the RBDs of SARS-CoV-2 with a K_D_ of 6.3 nM, whereas other antibodies such as m396, CR3014, and S230.15 failed to bind to the SARS-CoV-2-S protein [17,79]. However, a low level of binding to SARS-CoV-2-S was observed with a SARS-CoV-S1–specific polyclonal antibody T62 (#40150-T62, Sino Biological Inc., Beijing, China), and it could poorly neutralize SARS-CoV-2-S-protein–mediated virus entry. Further analysis revealed that the epitope for T62 was likely located on the RBDs of SARS-CoV-2-S, but detailed information is lacking [34]. In an exciting study, the Wilson laboratory determined the crystal structure of CR3022 antibody in complex with SARS-CoV-2-RBD (PDB: 6W41) and revealed that CR3022 binds a highly conserved epitope that is distantly located from the receptor-binding site, which enables cross-reactive binding, but could not neutralize SARS-CoV-2 in vitro [78] (Fig 5I). However, whether CR3022 can synergize with other SARS-CoV-2-RBD–binding antibodies for neutralization requires further evaluation and study.

The SARS-CoV (GenBank: AY278488.2) and SARS-CoV-2 (GenBank: MN908947.3) S proteins share about 76% amino acid sequence identity, suggesting that the remaining 24% amino acid sequences, which are nonconserved, might be responsible for antigenic differences between these 2 proteins. In the quest to find novel antibody binding epitopes on S proteins, Zheng and colleagues performed antibody epitope analysis and surface epitope accessibility using bioinformatic tools to identify both weak and strong epitopes, which might be otherwise experimentally ignored [80]. Their analysis identified 5 shared epitopes, along with 40 and 29 unique epitopes, on the S proteins of SARS-CoV and SARS-CoV-2, respectively. Among these unique epitopes, 92.7% originated from nonconserved regions, which might explain why most of the SARS-CoV–specific antibodies discussed in this review did not bind to the S protein of SARS-CoV-2 [80]. Taken together, these results suggest the necessity of developing SARS-CoV-2–specific antibodies and vaccine candidates.

### ACE2-independent receptors in viral pathogenesis

It is now established that both SARS-CoV and SARS-CoV-2 exploit hACE2 receptor to gain a host-cell entry [10,23]; however, some studies indicate that in addition to ACE2, SARS-CoV might exploit other host factors such as vimentin (a cytoskeleton protein) and lectins (a glycoprotein) to mediate viral entry [81,82]. It is important to note that the precise role of lectins in SARS-CoV infection has not been explored extensively, and the topic remains controversial. Jeffers and colleagues reported that SARS-CoV might use both ACE-2 and CD209L, a C-type lectin, to invade host cells [82]. In contrast, Zhou and colleagues and others indicated that mannose-binding lectins interfere with viral entry, potentially by blocking other interactions [83,84].

The S protein seems to be heavily glycosylated; however, the role of glycosylation in SARS-CoV-2 infection also remains an unexplored area. A recent in vitro study reported interactions between SARS-CoV-2 S protein and C-type lectins as well as sialic-acid binding lectins; however, a major limitation of this study is that it does not provide any cell-based in vivo data, and proper controls are missing [85]. SARS-CoV-2 seems to infect a diverse range of cell types; therefore, it is reasonable to speculate that ACE2-independent interactions might provide an alternate route for viral invasion. Given the importance of this topic and its massive impact on human lives, future studies will have to carefully evaluate whether non-ACE2 interactions compete with ACE2 to inhibit viral entry or ACE2-independent interactions produce a synergistic effect with ACE2-mediated entry to exacerbate the symptoms of COVID-19.

## Conclusions

The recent global outbreak of COVID-19 has killed almost 570,000 people (as of July 14, 2020) [7] and threatened the global economy, causing economic hardships to millions of people. Extensive progress has been made in terms of understanding the structure and function of the S glycoproteins. Specifically, decade-long structural studies on the S proteins of SARS-CoVs have designated 6 key residues (Y442, L472, N479, D480, T487, and Y491 for SARS-CoV) [67] in the RBDs that are critical for the host-cell ACE2 receptor binding, as well as for playing important roles in the cross-species transmission. Notably, 5 out of these 6 residues differ between the RBDs of SARS-CoV and SARS-CoV-2 S proteins, which have exhibited enhanced binding between the RBDs of SARS-CoV-2 and ACE2 receptors. This might be one of the reasons behind widespread human-to-human transmission of SARS-CoV-2. In addition, there are likely to be other factors that contribute to the infectivity and pathogenicity of SARS-CoV-2 that need to be investigated.

The trimeric prefusion structure of the SARS-CoV-2 S protein was obtained in an asymmetric conformation in which 1 protomer was observed in the “up” and the other 2 in the “down” conformations. This phenomenon, known as protein “breathing,” was observed in the S1 domain while determining the trimeric prefusion structure, which suggested the mechanism used by the CR3022 antibody to access a cryptic epitope on the trimeric S protein that is otherwise not possible. Interestingly, a similar breathing phenomenon identified unique and conserved epitopes in the trimeric interface of influenza hemagglutinin protein recently. The antibodies binding to these cryptic epitopes did not inhibit viral infection in vitro but conferred in vivo protection [86,87]. A similar phenomenon was observed in case of the CR3022 monoclonal antibody; therefore, further in vivo studies are required as soon as a suitable animal model is established for SARS-CoV-2 studies. In the course of writing this review, 2 exciting reports became available: (i) an antibody 47D11 that is reported to neutralize SARS-CoV-2, as well as SARS-CoV, in cell culture through an unknown mechanism that is different from the virus neutralization process [88]; and (ii) an inactivated novel coronavirus vaccine (PiCoVacc) that is able to induce SARS-CoV-2–specific neutralizing antibodies in mice, rats, and nonhuman primates. Additionally, data demonstrate that the PiCoVacc vaccine provides partial to complete protection in macaques against SARS-CoV-2 challenge [89]. Future investigations are required to understand the mechanism of neutralization by these antibodies.

Last but not the least, glycosylation has been an important measure of virus antigenic properties and plays a critical role for the manufacturing of effective vaccines against HIV and influenza. Notably, the SARS-CoV-2 S protein is densely decorated by host-derived heterogenous N-linked glycans, as indicated by a site-specific glycosylation analysis undertaken by mass spectrometry. Specifically, each SARS-CoV-2 S trimer displays 66 N-linked glycosylation sites with an elevation in oligomannose- and hybrid-type glycans compared with typical host-derived glycoproteins [90]. Finally, glycan profiling will be an important addition to measure antigen quality and should be examined while producing glycoprotein-based vaccine candidates for COVID-19.

Though it is observed that SARS-CoV-2 binds to its receptor on the host cells with higher affinities than SARS-CoV, the fatality rate caused by SARS-CoV-2 (3.4%) is significantly less than the reported rate of SARS-CoV (9%–11%, WHO). The reasons behind these differences remain elusive, and future research will shed light on these variations. Recent sequencing data indicate that the SARS-CoV-2 mutation rate is approximately 25 mutations per year. If these mutations enable more efficient virus spread and increased pathogenicity, then vaccine development will be a challenging task. Hopefully, future studies will be able to resolve these questions and come up with medications as well as vaccines against this deadly virus. Even with the vaccine and medications against this virus, future outbreaks of similar viruses and pathogens are likely to continue. Therefore, apart from curbing this outbreak, government policies and efforts should be made to formulate thorough measures to prevent future outbreaks of viruses and bacteria (there is already a significant threat from antibiotic-resistant bacteria).

## Supporting information

S1 Fig Phylogenetic tree of coronaviruses. The figure shows the phylogenetic tree drawn for 69 coronavirus genomic sequences, including the SARS-CoV-2 sequences. Sequences belonging to different Coronavirinae subfamilies are labeled. SARS-CoV-2, Severe Acute Respiratory Syndrome Coronavirus-2.
(TIF)
