# Supplementary figures and images for: COVID-19 pandemic: Insights into structure, function, and hACE2 receptor recognition by SARS-CoV-2
Source: PLoS Pathog. 2020 Aug 21;16(8):e1008762. doi: 10.1371/journal.ppat.1008762 (PMC7444525; doi:10.1371/journal.ppat.1008762)

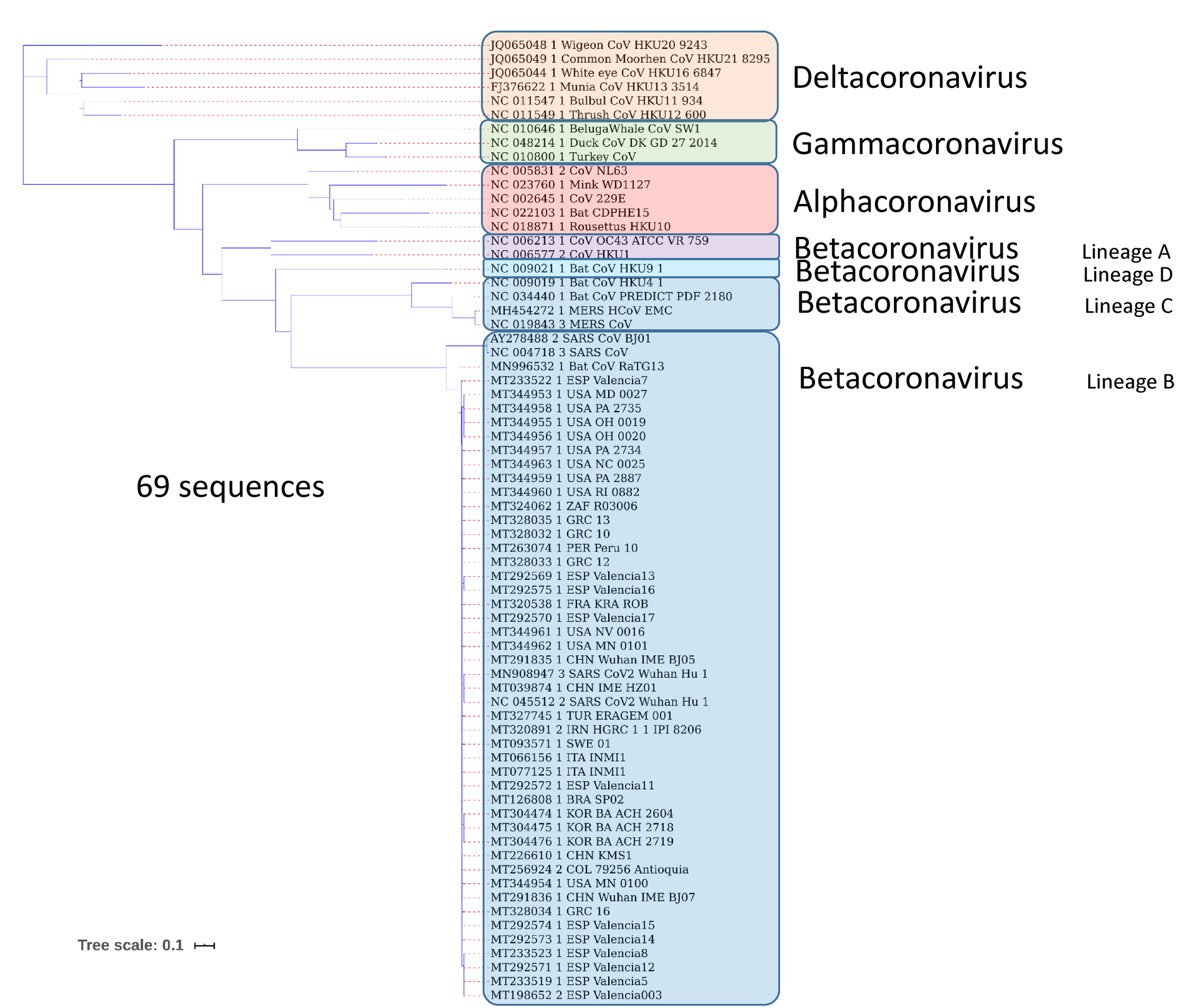

Supplement: S1 Fig — The figure shows the phylogenetic tree drawn for 69 coronavirus genomic sequences, including the SARS-CoV-2 sequences. Sequences belonging to different Coronavirinae subfamilies are labeled. SARS-CoV-2, Severe Acute Respiratory Syndrome Coronavirus-2. (TIF) [file ppat.1008762.s001.tif]
